# RNA sequencing data of human prostate cancer cells treated with androgens

**DOI:** 10.1016/j.dib.2019.104372

**Published:** 2019-08-09

**Authors:** Raghavendra Tejo Karthik Poluri, Charles Joly Beauparlant, Arnaud Droit, Étienne Audet-Walsh

**Affiliations:** a Department of Molecular Medicine, Faculty of Medicine, Université Laval, Axe Endocrinologie – Néphrologie du Centre de recherche du CHU de Québec – Université Laval, Québec City, Québec, Canada; b Centre de recherche sur le cancer de l'Université Laval, Québec City, Québec, Canada

**Keywords:** Steroid, Nuclear receptor, Hormone receptor, Metabolism, Metabolic reprogramming, Castration-resistance, Mitochondria, Glycolysis, Fatty acid metabolism

## Abstract

Prostate cancer (PCa) is the most frequent cancer in North American men and PCa cells rely on the androgen receptor (AR) for growth and survival. To understand the effect of AR in cancer cells, we have treated LNCaP and LAPC4 cells, two immortalized human PCa cells *in vitro*, with the synthetic androgen R1881 and then performed RNA-seq analyses. High quality sequencing data have been analyzed using our bioinformatic pipeline which consists of FastQC for quality controls, Trimmomatic for trimming, and Kallisto for pseudoalignment to the transcriptome. Differentially expressed genes were identified using DESeq2 after adjustment for false-discovery rate (FDR *q* values < 0.05) and Relative Log Expression (RLE) normalization. Gene Set Enrichment Analysis (GSEA) was also performed to identify biological pathways significantly modulated by androgens. GSEA analyses identified the androgen signaling pathway, as well as several metabolic pathways, as significantly enriched following androgen stimulation. These analyses highlight the most significant metabolic pathways up-regulated following AR activation. Raw and processed RNA-seq data were deposited and made publicly available on the Gene Expression Omnibus (GEO; GSE128749). These data have been incorporated in a recent article describing the functions of AR as a master regulator of PCa cell metabolism. For more details about interpretation of these results, please refer to “Functional genomics studies reveal the androgen receptor as a master regulator of cellular energy metabolism in prostate cancer” by Gonthier et al. (doi: 10.1016/j.jsbmb.2019.04.016).

**Table:** Specifications Table

Subject area	Cancer Research, Endocrinology, Androgen receptor
More specific subject area	Prostate Cancer, Molecular Biology, Bioinformatics, Cancer Genomics, Steroid, castration-resistant, androgen
Type of data	*Transcriptomic data*
How data was acquired	RNA-sequencing (125bp paired end sequencing using a HiSeq 2500)
Data format	*Raw and processed RNA-seq data. Raw data (FASTQ) and processed RNA-seq data, including TPM and FPKM values, are fully available.*
Experimental factors	*Cells were treated with* 10nM *of the synthetic androgen R1881 or vehicle (ethanol 96%)*
Experimental features	*Cells were seeded in RPMI 1640 media with no phenol red and containing 5% charcoal-stripped serum for 48h to allow steroid deprivation. Media was then changed, and fresh media with* 10nM *R1881 or vehicle (EtOH 96%) was added. After 24h treatment, cells were harvested and RNA was purified with RNeasy super purification kit from QIAGEN. RNA was then sent to the Genomic Centre of the Centre de recherche du CHU de Québec - Université Laval for mRNA enrichment and RNA-sequencing. Standard protocol of NEBNext Ultra II Directional RNA library prep kit was followed for mRNA enrichment and library preparation.*
Data source location	Quebec City, Quebec, Canada
Data accessibility	Both raw and processed RNA-seq data were deposited on the Gene Expression Omnibus (GEO) and made publicly available (GSE128749). https://www.ncbi.nlm.nih.gov/geo/query/acc.cgi?acc=GSE128749
Related research article	Functional genomics studies reveal the androgen receptor as a master regulator of cellular energy metabolism in prostate cancer by Gonthier et al. (https://doi.org/10.1016/j.jsbmb.2019.04.016)

**Table:** 

**Value of the data** • Bioinformatic analyses of differentially expressed genes and biological pathways regulated by androgens can be studied for a better understanding of the effect of AR in PCa. • Validation in two distinct PCa cell lines allow for the identification of more reproducible results. • These data highlight a new function of AR in PCa as a master regulator of cellular energy metabolism. • These data may allow the discovery of new therapies targeting the unique PCa cell metabolic program.

## 1 Data

The raw data (.fastq files) generated from Illumina sequencing were deposited on the Gene Expression Omnibus (GEO) with the reference number GSE128749 (https://www.ncbi.nlm.nih.gov/geo/query/acc.cgi?acc=GSE128749). The comma separated value files (.csv) which have been produced after the quantification and pseudoalignment with the transcriptome hg38 using Kallisto were also uploaded on GEO. These files contain the raw counts, the transcripts per million (TPM) values, and the fragments per kilobase million (FPKM) values for every sample. Differentially expressed genes on normalized data were identified using a FDR *q* value < 0.05.

## 2 Experimental design, materials, and methods

### 2.1 Cells

LNCaP and LAPC4, two androgen receptor (AR) positive human PCa cell lines, were initially obtained from the ATCC and re-authentified in 2016 [1]. After resuscitation, the cells were not kept in culture for more than 3 months. Cells were grown in RPMI 1640 supplemented with 10% fetal bovine serum (FBS), streptomycin, penicillin, and sodium pyruvate in 37 °C incubators with 5% CO_2_. Before androgen stimulation, cells were trypsinized and seeded at a 70% confluence in RPMI-1640 media with no phenol-red and supplemental with 5% charcoal-stripped serum (CSS), streptomycin, penicillin, and sodium pyruvate, as described previously [2]. After hormonal deprivation (48h), media was changed and fresh media containing 10nM of the synthetic androgen R1881 or vehicle (EtOH 96%). 24h later, cells were harvested for RNA purification using the RNA purification kit RNeasy plus mini kit from QIAGEN.

### 2.2 Sequencing

Excellent RNA integrity was confirmed using a TapeStation 2200 (Agilent); all samples had an RNA integrity number equivalent (RIN^e^) > 8.5. mRNA enrichment and library preparation were performed using the NEBNext Ultra II Directional RNA library prep kit following the manufacturer's protocol. RNA was then sent to the Genomic Centre of the Centre de recherche du CHU de Québec - Université Laval for sequencing using a HiSeq 2500 (125bp paired-end sequencing).

### 2.3 RNA-seq analysis

After sequencing, raw data were obtained in the fastq format. FastQC [3] was used for validating the quality of the data. Trimming of the adaptor content and over-represented sequences was performed using Trimmomatic [4]. Also note that trimming was performed with the minimal length (MINLEN) set at 36. Quality check using FastQC was performed again on the trimmed sequences (Table 1). For the pseudoalignment of the trimmed sequences to the hg38 transcriptome, the Kallisto tool was used [5]. Final normalization was performed using the Relative Log Expression (RLE) method [6]. We have used the R-package called Tximport to convert the transcript quantifications to gene quantifications [7].

**Table 1:** Number of reads for raw and trimmed sequences of PCa cells treated with androgens.

Cell lines	Treatment	Reads (Raw)	Reads (after trimming)
LNCaP	Control #1	9327182	7694732
	Control #2	11058265	9014786
	Control #3	10258931	8909738
	R1881 #1	7812714	6616969
	R1881 #2	9964804	8508748
	R1881 #3	10496859	8965255
LAPC4	Control #1	8744802	7390781
	Control #2	6528343	5443146
	Control #3	10146342	8641030
	R1881 #1	9384625	7940193
	R1881 #2	11134285	9474978

### 2.4 Differential gene expression and GSEA analysis

**Fig. 1:**
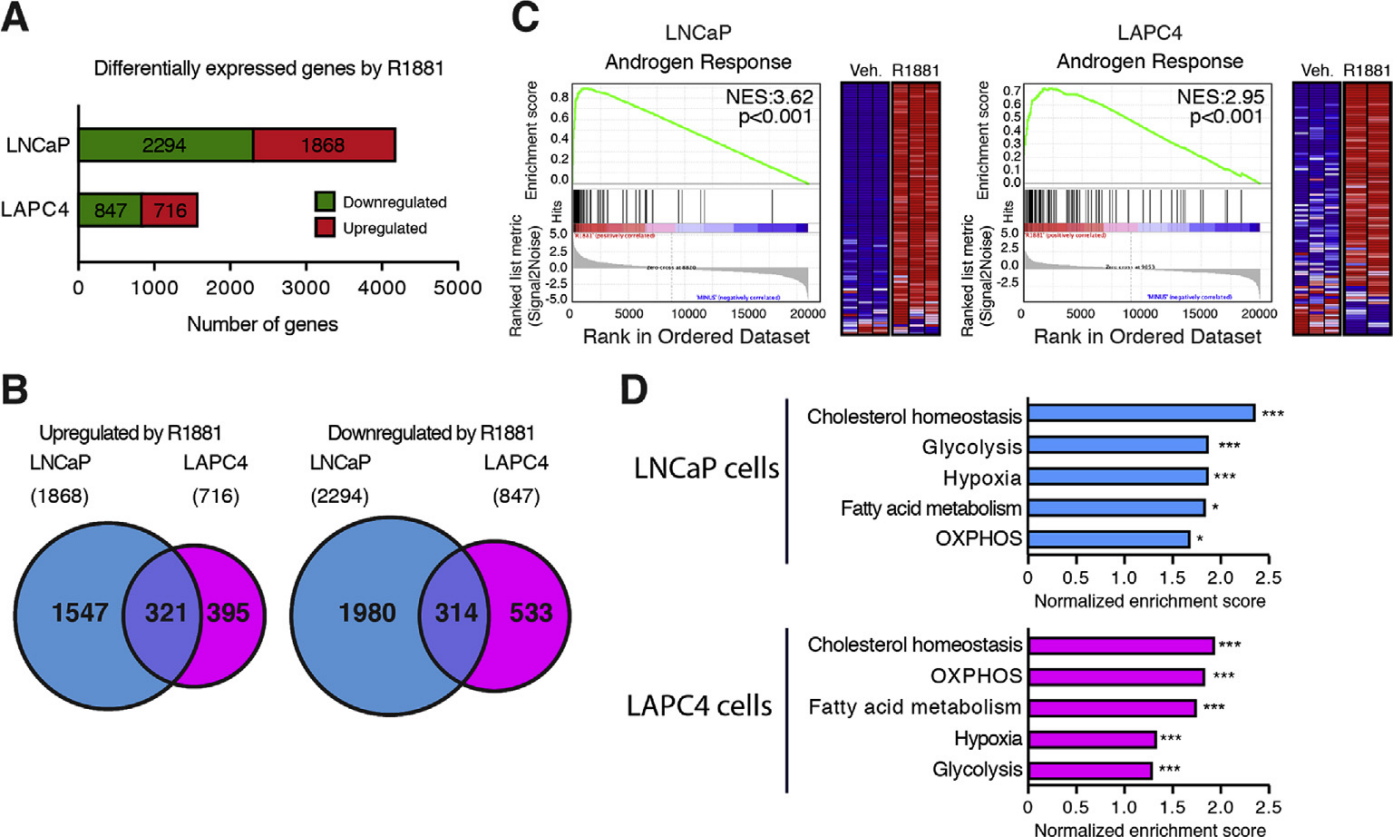
Transcriptomic analyses of the androgen signaling pathway functions in human prostate cancer cells. A) Number of genes significantly up- or down-regulated following treatment with R1881 in LNCaP and LAPC4 cells. A FDR *q* value < 0.05 was used to identify differentially expressed genes. B) Venn diagrams showing the overlap between genes up-regulated (left) and down-regulated (right) by R1881 in LNCaP and LAPC4 cells. C) Gene set enrichment analysis (GSEA) plots for the “Hallmarks - Androgen Response” signature in LNCaP and LAPC4 cells. NES: normalized enrichment score. D) GSEA signatures enrichment scores for significantly enriched metabolic pathways in LNCaP and LAPC4 cells following 24h treated with R1881. OXPHOS: oxidative phosphorylation (mitochondrial respiration) *p < 0.05; **p < 0.01; ***p < 0.001.

To study genes regulated by AR in PCa cells, differential expressed genes were identified using a FDR *q* value < 0.05 with DESeq2 [8]. Overall, 1868 and 716 genes were up-regulated in LNCaP and LAPC4 cells and 2294 and 847 genes were significantly down-regulated in LNCaP and LAPC4 cells, respectively (Fig. 1A). Of these, 321 common genes were up-regulated while 314 common genes were down-regulated in both cell lines (Fig. 1B). GSEA analyses [9] were also performed using TPM values to identify the most significantly up-regulated pathways following activation of AR in these human PCa cells. In both cell lines, the androgen signaling pathway was highly enriched following R1881 treatment (Fig. 1C). In addition, several metabolic pathways were also enriched in both LNCaP and LAPC4 cells following AR activation (Fig. 1D).

## References

[1] Audet-Walsh E. (2017). Androgen-dependent repression of ERRgamma reprograms metabolism in prostate cancer. Cancer Res..

[2] Audet-Walsh E. (2017). Nuclear mTOR acts as a transcriptional integrator of the androgen signaling pathway in prostate cancer. Genes Dev..

[3] Andrews S. (2010). FastQC A Quality Control Tool for High Throughput Sequence Data. https://www.bioinformatics.babraham.ac.uk/projects/fastqc/.

[4] Bolger A.M., Lohse M., Usadel B. (2014). Trimmomatic: a flexible trimmer for Illumina sequence data. Bioinformatics.

[5] Bray N.L. (2016). Near-optimal probabilistic RNA-seq quantification. Nat. Biotechnol..

[6] Anders S. (2013). Count-based differential expression analysis of RNA sequencing data using R and Bioconductor. Nat. Protoc..

[7] Soneson C., Love M.I., Robinson M.D. (2015). Differential analyses for RNA-seq: transcript-level estimates improve gene-level inferences. F1000 Res.

[8] Love M.I., Huber W., Anders S. (2014). Moderated estimation of fold change and dispersion for RNA-seq data with DESeq2. Genome Biol..

[9] Mootha V.K. (2003). PGC-1alpha-responsive genes involved in oxidative phosphorylation are coordinately downregulated in human diabetes. Nat. Genet..

